# Ultrafast bursts of tailored spatiotemporal vortex pulses

**DOI:** 10.1038/s41377-025-02062-y

**Published:** 2025-10-10

**Authors:** Xin Liu, Chunhao Liang, Qian Cao, Yangjian Cai, Qiwen Zhan

**Affiliations:** 1 School of Optical-Electrical and Computer Engineering, University of Shanghai for Science and Technology, Shanghai, 200093 China; 2 Shandong Provincial Engineering and Technical Center of Light Manipulations and Shandong Provincial Key Laboratory of Optics and Photonic Device, School of Physics and Electronics, Shandong Normal University, Jinan, 250014 China; 3 Collaborative Innovation Center of Light Manipulations and Applications, Shandong Normal University, Jinan, 250358 China; 4 Zhejiang Key Laboratory of 3D Micro/Nano Fabrication and Characterization, Department of Electronic and Information Engineering, School of Engineering, Westlake University, Hangzhou, Zhejiang 310030 China; 5 Zhangjiang Laboratory, 100 Haike Road, Shanghai, 201204 China; 6 International Institute for Sustainability with Knotted Chiral Meta Matter (WPI-SKCM2), Hiroshima University, Higashihiroshima, Hiroshima 739-8526 Japan

**Keywords:** Ultrafast photonics, Ultrafast photonics

## Abstract

Orbital angular momentums (OAMs) of light can be categorized into longitudinal OAM (L-OAM) and transverse OAM (T-OAM). Light carrying time-varying L-OAM, known as self-torqued light, was recently discovered during harmonic generation and has been extensively developed within the context of optical frequency combs (OFCs). Meanwhile, ultrafast bursts of optical pulses, analogous to OFCs, are sought for various light-matter interaction, spectroscopic and nonlinear applications^1–6^. However, achieving transiently switchable T-OAM of light on request, namely spatiotemporal vortex pulse bursts, with independently controlled spatiotemporal profile of each comb teeth, remains unrealized thus far. In this work, the experimental generation of spatiotemporal vortex bursts featured with controllable time-dependent characteristics is reported. The resultant bursts comprised of spatiotemporal optical vortex comb teeth have picosecond timescale switchable T-OAMs with defined arrangement. We also show ultrafast control of T-OAM chirality, yielding pulse bursts with staggered azimuthal local momentum density, resembling Kármán vortex streets. This approach enables the tailoring of more intricate spatiotemporal wavepacket bursts, such as high-purity modes variation in both radial and azimuthal quantum numbers of spatiotemporal Laguerre-Gaussian wavepackets over time, which may facilitate a host of novel applications in ultrafast light-matter interactions, high-dimensional quantum entanglements, space-time photonic topologies as well as spatiotemporal metrology and photography.

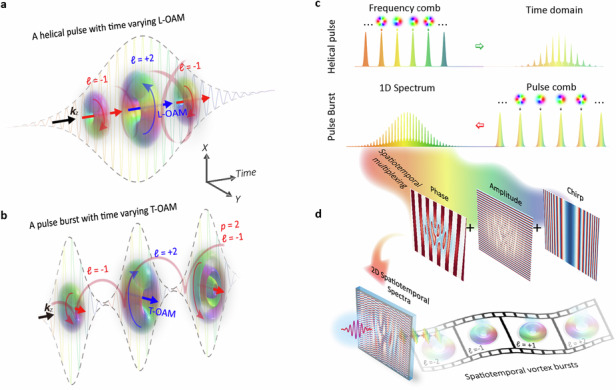

## Abstract (web summary)

A spatiotemporal vortex pulse burst, akin to an optical comb, with controllable time-dependent characteristics is reported.

## Introduction

**Fig. 1:**
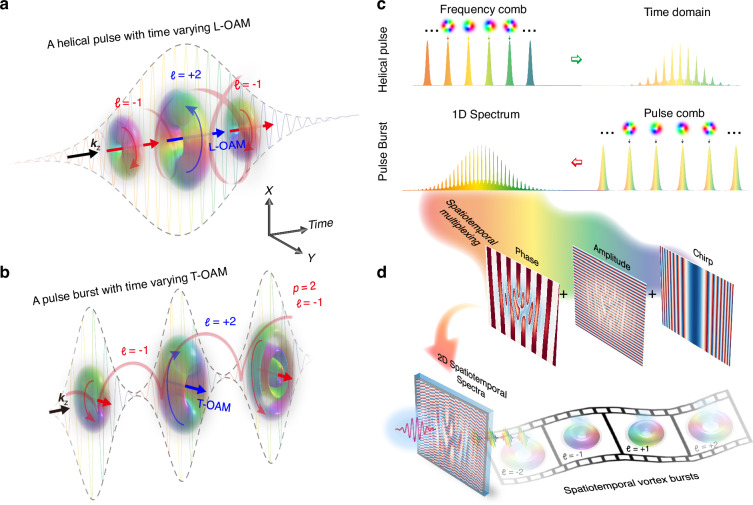
**Conceptual scheme of the synthesis of spatiotemporal vortex bursts with time-varying dynamic properties**. **a** Time-varying L-OAMs of helical pulse. **b** Time-varying T-OAMs of spatiotemporal vortex bursts. **c** Analogy between optical frequency combs and pulse bursts. **d** Principle for spatiotemporal vortex bursts generation by engineering the 2D spatiotemporal spectra (Methods)

Finite trains of ultrashort pulses, commonly referred to as pulse bursts and similar to optical frequency combs consisting of a series of separated “comb teeth” of frequencies, have already been utilized in various applications, including laser materials processing^1,2^, nonlinear science^3,4^, ultrafast photography^5,6^, among many others. In recent years, new research avenues have been opened up by exploring two-dimensional spatiotemporally structured light with desired attributes. Spatiotemporally coupled wavepackets with diverse geometric and topological textures exhibit unique physical properties during propagation, focusing, and interaction with matter^7^. However, versatile pulse bursts comprising spatiotemporal wavepackets with tailored photon degrees of freedom (DoFs) have not been reported^8,9^. Orbital angular momentum (OAM) as a promising DoF of light is associated with its twisted waveform, resulting in phase singularities surrounded by a symmetric doughnut intensity profile^10^. The photonic intrinsic (coordinates-independent) OAM can be either longitudinal (L-OAM) if it arises from a helical phase in the spatial domain or transverse (T-OAM) if it emerges in the space-time domain^11–14^. The L-OAM was early discovered in spatial optical vortices by Allen et al.^15^, which has been extensively studied and has spurred numerous significant applications^16,17^. The T-OAM is a newly discovered property of photons in spatiotemporal optical vortices (STOVs), as demonstrated both theoretically^18,19^ and experimentally^20–23^. This revolutionary progress propels research toward nonlinear optics^24–26^, novel photonics topology^27,28^, spatiotemporal wavepackets engineering^29–31^ and other matter waves^32–34^, etc. Spatiotemporal structure wavepackets featuring controllable multiple photon DoFs have also been experimentally constructed^35^. While a conventional spatial/spatiotemporal vortex beam generally carries a temporally static OAM. In this context, self-torqued beam with time-varying OAM was first created in a high harmonic generation driven by two ultrafast pulses with different OAM and time delayed to each other^36^. Since then, spatiotemporal wavepackets carrying time-dependent OAM have been sequentially synthesized, remarkably in ultrafast light coils through correlating spatial modes with time frequencies^37–40^, tailored spatiotemporal dynamic wavepackets by means of a multi-plane light conversion^41,42^, and integrated vortex emitter by imprinting OAM modes into frequency microcomb^43,44^. However, to date, the majority of dynamic spatiotemporal beams are primarily restricted to helical wavepackets, that is time-varying L-OAMs along light pulse, as shown in Fig. 1a.

Researchers recently observed subtle changes in T-OAM density by disrupting an STOV pulse^45^. By intentionally offsetting phase singularities along the frequency, time-separated singularities can be generated through a linear mapping^46,47^. However, efforts have primarily focused on controlling and shaping a single STOV pulse, where the manipulation of DoFs on demand is limited and frequency-dependent, and the resulting space-time distortions compromise T-OAM purity and introduce significant crosstalk between singularities. In this context, generating a set of isolated STOV pulse bursts with arbitrary time-dependent photon properties presents a promising solution but has yet to be demonstrated. In this work, we achieve an ultrafast pulse train of independent spatiotemporal Laguerre-Gaussian (STLG) basis with variable quantum numbers, constructing “spatiotemporal vortex bursts” with tailored time-varying DoFs (Fig. 1b), that resemble OFCs. Instead of conventional spatial modes^41,42^, each comb teeth within the burst lies on the space-time plane in a spatiotemporally coupled manner. The magnitude and arrangement of radial and azimuthal quantum numbers printed on each comb teeth can be self-defined on demand. The resulting spatiotemporal vortex bursts appear to resemble flying donuts with ultrafast switchable quantum numbers, reaching terahertz intraburst repetition rate conversion between high-purity mode indices.

## Principle and experiment

**Fig. 2:**
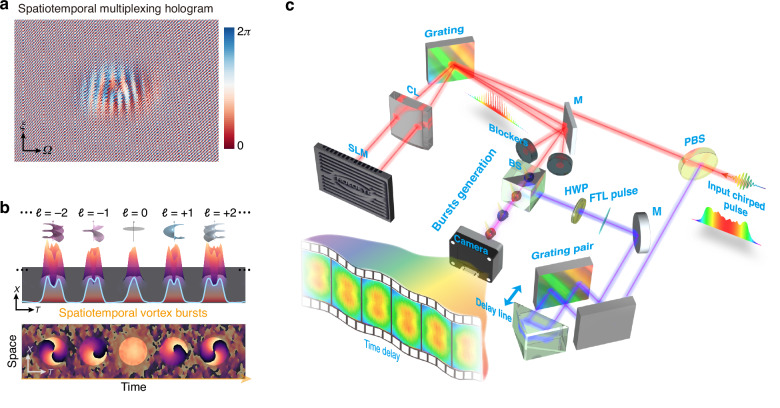
**Generation of ultrafast pulse burst of STOV wavepacket based on spatiotemporal multiplexing technique**. **a** Pattern of a spatiotemporal multiplexing hologram consisting of a train of five STOV pulses, with topological charges ranging from *ℓ* = −2 to +2 as an example. **b** The light modulated by (**a**) is demultiplexed in the time domain, forming an STOV pulse comb. **c** Experimental configurations for tailored spatiotemporal vortex bursts generation and measurement. PBS polarization beam splitter, CL cylindrical lens, SLM spatial light modulator, M mirror, BS beam splitter, HWP half-wave plate

The conceptual scheme for the synthesis of spatiotemporal vortex bursts is illustrated in Fig. 1c, d. Optical pulse bursts and frequency combs can be viewed as inverse processes in the time-frequency domain. By correlating spatial vortices with finite comb teeth within an OFC, self-torqued helical pulse with time-varying L-OAMs can be generated in the time domain^43,44^. Conversely, an optical pulse burst exhibits a pulse comb in the time domain resulting in a complex spectrum like the temporal representation of such OFC (Methods), as shown in Fig. 1c. Therefore, to synthesize a pulse burst comprising a chain of regular spatiotemporal vortex pulses, we assign specific spatiotemporal coupling mode into each comb teeth in the time domain, forming a complex 2D spatial-spectral representation (Fig. 1d). The complex representation of Eq. (5) is encoded into a spatiotemporal multiplexing hologram (Methods), exemplified in Fig. 2a, which allows fully tailoring the individual STOV comb teeth with the same central frequency. By implementing a 2D space-time Fourier transform (FT) on this hologram, the zero-order output is demultiplexed in the space-time domain, generating the desired spatiotemporal vortex burst, as illustrated in Fig. 2b. Each comb teeth corresponds to an isolated and well-defined STOV pulse with time-dependent characteristics, and its spatiotemporal profile can be arbitrarily tailored by designing the corresponding spatiotemporal multiplexing hologram. The experimental configurations utilized in our work are based on the integration of a 2D ultrafast pulse shaper and spatiotemporal multiplexing technique, as depicted in Fig. 2c. In the experiment, the hologram mask displayed on a reflective phase-only spatial light modulator is inserted in the intermediate confocal plane of a 4f ultrafast pulse shaper. With the help of a grating and a cylindrical lens, the spatial-spectral distribution of an incident pulse can be readily tailored by this mask. In our setup, the temporal FT was achieved via a grating and a cylindrical lens, the spatial FT was achieved via free-propagation after the pulse shaper (around 1.2 m). Since all comb teeth within a burst are spatiotemporally coherent, we can use a dechirped reference beam to interrogate their 3D spatiotemporal morphology (see Supplementary Note 1 for the detailed experiment principle). The intraburst repetition rate and order inside the synthesized spatiotemporal vortex burst are determined by the intraburst phase slips. In addition to imparting the T-OAM content, a group delay dispersion phase is added to chirp the pulse for balancing spatial diffraction and temporal dispersion, which is essential to produce a high mode purity spatiotemporal vortex burst (Supplementary Note 2).

## Results

**Fig. 3:**
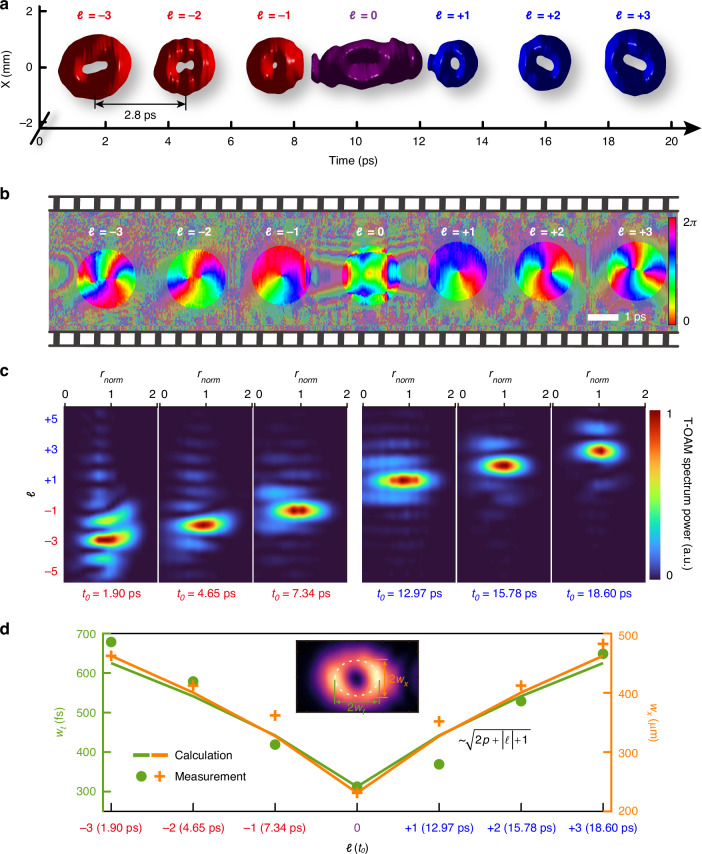
**Synthesized spatiotemporal vortex burst with time-varying T-OAM from** ***ℓ*** = −3 to +3. **a** Measured iso-intensity profile of the spatiotemporal vortex burst in the space-time domain, which has a ~20 ps temporal length and a 2.8 ps temporal pitch. The isovalue is set to 8.5% of the peak intensity. The red and blue color denotes the negative and positive *ℓ* respectively. **b** Retrieved phase distribution in the meridional plane. **c** T-OAM modal analysis of the synthesized spatiotemporal vortex bursts, exhibiting time-varying T-OAMs with high mode purity (Supplementary Fig. S3), where ${r}_{{\rm{norm}}}=\sqrt{{\left(T-{t}_{0}\right)}^{2}/{w}_{t}^{2}+{(X-{x}_{0})}^{2}/{w}_{x}^{2}}$ is a normalized spatiotemporal polar radius (Supplementary Note 3). **d** Measured temporal/spatial radius (${w}_{t}$ and ${w}_{x}$) as a function of *ℓ* (${t}_{0}$), showing a spatiotemporal angular deceleration and acceleration along time, indicating an angular deceleration/acceleration on the space-time plane

We firstly generate a spatiotemporal vortex burst with time-varying azimuthal quantum numbers from *ℓ* = −3 to +3 in Fig. 3. Such spatiotemporal vortex burst is designed as consisting of 7 LG bases of radial index *p* = 0 with a temporal pitch of 2.8 ps. Figure 3a presents the 3D iso-intensity profile of the synthesized burst in the space-time domain, reconstructed from 600 temporal slices from 0 ps to ~20 ps in the experiment (Supplementary Note 1 and Supplementary Movie 1). The measured iso-surface showcases a pulse comb consisting of a series of STOV with variable sizes, each separated from the adjacent ones by 2.8 ps. Each comb teeth exhibits a central dark hole induced by their spatiotemporal phase singularities, as shown in Fig. 3b. The phase distribution also confirms the time-varying azimuthal quantum numbers from *ℓ* = −3 to +3. The T-OAM spectrum power distributions (Supplementary Note 3) of the synthesized spatiotemporal vortex burst are shown in Fig. 3c. Each comb teeth exhibits high mode purity at its desired mode index (Supplementary Fig. S3). The measured bursts present a temporal evolution of T-OAM and appear as an angular acceleration/deceleration on the space-time plane, as plotted in Fig. 3d, which implies a terahertz variation $\partial {\mathcal{l}}({\theta }_{x-t})/\partial t \sim 0.36\,{\rm{THz}}$ of T-OAMs. Such an ultrafast variation in mean T-OAM avoids intermediate OAM states induced by modal interference, as the individual pulses within the burst remain isolated. This enables a straightforward and ultrafast approach to controlling their temporal properties.

**Fig. 4:**
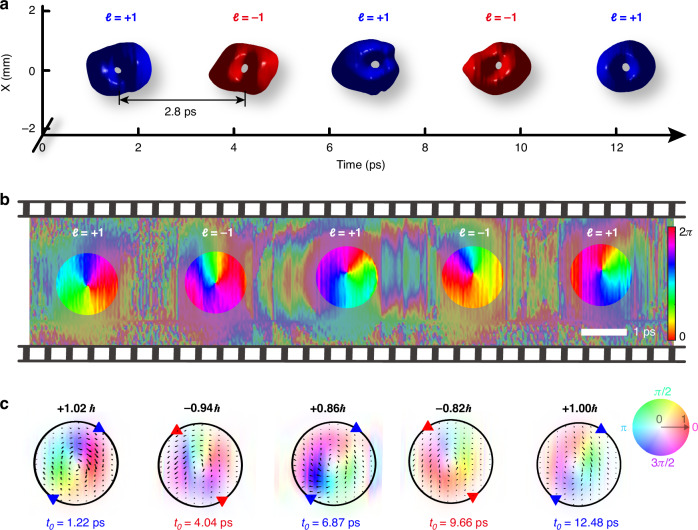
**Burst of spatiotemporal vortex pulses with time-varying chirality alternating between** ***ℓ*** = **+1 and** ***ℓ*** = −1. **a** Measured iso-intensity profile. The isovalue is set to 11% of the peak intensity. **b** Retrieved phase in the meridional plane. **c** Complex field and energy density flux (calculated with ${\beta }_{2}=-{k}_{0}^{-1}$, see Supplementary Note 3 and Note 5) of pulse burst in the space-time domain, reminiscent of the KVS featured temporal variation of chirality of T-OAM. The values at the top denote the mean value of the T-OAM $\left\langle {L}_{y}\right\rangle$ per photon for each comb teeth. The saturation and hue denote amplitude and phase information respectively

The proposed scheme enables easy customization of spatiotemporal vortex bursts with self-defined arrangement of T-OAMs. To illustrate this capacity, we generate a spatiotemporal vortex burst involving a series of spatiotemporal vortices with alternating azimuthal quantum numbers between *ℓ* = +1 and *ℓ* = −1. Figure 4a shows the measured 3D iso-intensity (at 11% of the peak intensity) profile of this spatiotemporal vortex burst, which includes 5 STOVs with 2.8 ps temporal pitch within a total temporal span of 12 ps. Each comb teeth features an independent dark hole, resulting in a flying doughnut-shaped appearance. The spatiotemporal evolution of intensity and phase of the bursts can be easily demonstrated by their time-dependent interfered fringes (Supplementary Note 4). Figure 4b presents the phase distribution of the generated burst, showing the temporally alternating variation of *ℓ* between +1 and −1. To further elucidate the local spatiotemporal distributions of optical energy inside the burst, we plot the complex field and corresponding energy density flux (calculated using anomalous dispersion coefficient ${\beta }_{2}=-{k}_{0}^{-1}$, see Supplementary Note 5 for detail) in Fig. 4c. Over time, the energy density flux exhibits “spiral” patterns circulating around the corresponding singularities (with opposite circulations, namely a vortex dipole), with the mean T-OAM per photon equals to the topological charge carried by each comb teeth. All results indicate the staggered reversal of energy chirality along time, forming an optical analogy to the Kármán vortex street (KVS). This phenomenon, characterized by the alternating chirality of swirling vortices, is caused by the nonlinear process of vortex shedding in fluid dynamics and has also been predicted in the linear realm^48^ (Supplementary Note 6). Moreover, whereas KVS in fluid dynamics is inherently a nonlinear phenomenon, here we demonstrate that analogous effects can arise in a polychromatic wavepacket propagating at the group velocity under conditions of linear anomalous dispersion. The instantaneous temporal variation of the T-OAM in a pulse burst is expected to play a role in the light-matter interactions, unveiling their energy transfer^49,50^. We can actively adjust the temporal separation and order of azimuthal quantum numbers within a burst, displaying self-defined time-varying T-OAMs (Supplementary Note 7).

**Fig. 5:**
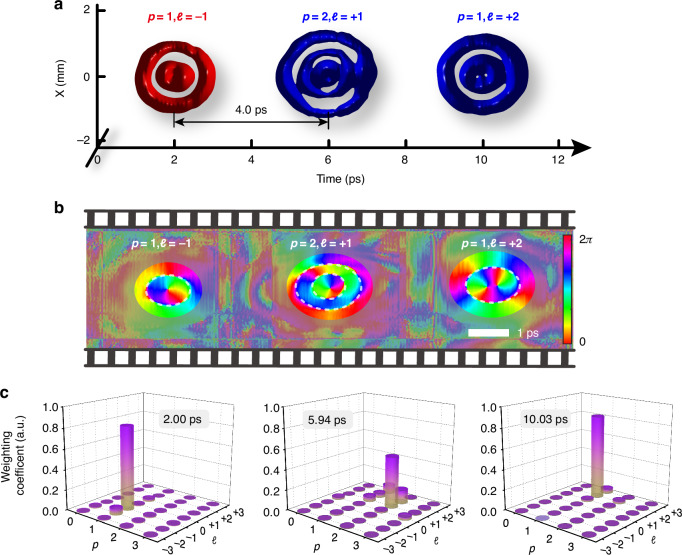
**Burst of spatiotemporal vortex pulses with defined time-varying quantum numbers on both radial and azimuthal indices**. **a** Measured iso-intensity profile of this spatiotemporal vortex bursts, exhibiting a train of time-varying STLG wavepackets with variable *p* and *ℓ*. The temporal separation is 4 ps. The isovalue is set to 5% of peak intensity. **b** Retrieved phase distribution in the meridional plane, showing time-varying spatiotemporal phase singularities and edge dislocations. **c** Modal weight analysis of each comb teeth at specific times within the burst, the power distributing at the desired radial ($p$) and azimuthal (${\mathcal{l}}$) index is 82.5%, 51.34%, and 84.4%, respectively

Beyond the time-dependent azimuthal quantum numbers shown, our scheme can be readily reconfigured on demand to create more elaborate spatiotemporal dynamics. To further illustrate this versatility, we generate a spatiotemporal wavepacket burst involving three STLG wavepackets, a set of complete and orthonormal basis, each assigned to a distinct azimuthal (*ℓ*) and radial (*p*) quantum number. Figure 5a shows the measured 3D iso-intensity profile of the synthesized STLG wavepacket burst, which involves 3 STLG wavepackets ($p=1\,\&\, l=-1$, $p=2\,\&\, l=+1$ and $p=1\,\&\, l=+2$) with 4 ps temporal pitch within a temporal span of 12 ps. The corresponding spatiotemporal phase distribution is plotted in Fig. 5b. This burst shows dynamic spatiotemporal phase singularities and edge dislocations along time, resulting in time-varying coupled multi-ring topologies. Figure 5c shows the modal analysis for each comb teeth of this burst (Supplementary Note 8). The power distribution located at the desired radial and azimuthal index is examined to be 82.5%, 51.34 and 84.4% respectively at the time ${t}_{0}\approx$ 2 ps, 6 ps and 10 ps, clearly confirming the generation of high-purity time-varying STLG wavepackets. This involves carving dual time-varying DoFs of light field in a pulse burst. It is worth mentioning that the mode purity degradation of STLG modes with larger radial quantum numbers *p* arises from imperfections of the experimental generation. The mismatch between the ideal and experimental STLG wavepackets becomes more pronounced as the radial quantum number increases, further amplifying mode purity degradation (Supplementary Note 8). Additionally, we can achieve the spatiotemporal collision of dual STLG wavepackets inside a burst by controlling the relative delay between comb teeth, leading to more complex time-varying spatiotemporal properties (Supplementary Note 9).

## Discussion

In summary, we have demonstrated a new type of spatiotemporal vortex bursts featured by tailored spatiotemporal coupling properties at variable temporal locations. The generated spatiotemporal vortex bursts consist of tailored spatiotemporal vortex wavepackets, exhibiting temporally varied T-OAMs at picosecond timescale. Our scheme achieves temporal variation with self-defined arrangements on both the radial and azimuthal quantum numbers of STLG wavepackets. Our results enhance the ability to precisely control ultrashort pulses in both time and space simultaneously. The methodology demonstrated here could be further developed to tailor more sophisticated spatiotemporal structure wavepackets with tunable dynamic properties^41,51^ (Supplementary Note 11). The preparation of spatiotemporal vortex bursts is of great importance for revealing T-OAM-matter interactions in physicochemical processes, space-time photonic topologies, multiplexed optical communications and ultrafast events probing, etc. The pulse burst with time-varying tailored spatiotemporal properties enables the potential to realize higher-dimensional topological quasiparticles^52^. Although the bulky elements with a limited resolution in our demonstrations hamper the number of pulses per burst (Supplementary Note 12), employing advanced metasurface devices could further significantly enhance modulation efficiency, enable ultra-high resolution and ultra-high bandwidth for spatiotemporal light control^53,54^.

In our study, the STOV comb teeth within the pulse burst are isolated and spatiotemporally separated, meaning they are orthogonal to each other in both spatial and temporal coordinates. As a result, in nonlinear interactions, the pulses act individually in various nonlinear processes, such as sum-frequency generation, difference-frequency generation and second- or high-order harmonic generation^24–26,55^. The STOV comb, which carries identical central frequencies, might enable precise synchronization of interacting comb teeth, optimizing the efficiency of nonlinear processes. Moreover, individual pulse within the ultrafast burst carries customized space-time coupling properties, making them potential drivers for novel electron acceleration and radiation sources^9,56,57^. Additionally, the high repetition rate and tailored spatiotemporal characteristics of these pulse bursts provide a new time-dependent DoF, enabling promising applications in micromachining^1,2^, nonlinear spectroscopy^3,58^ and laser wakefield acceleration^4,59,60^. On the other hand, the STOV pulse bursts are spatiotemporally structured polychromatic fields, their propagation is governed by the interplay between spatial diffraction and temporal dispersion^7^. The electromagnetic energy flux associated with the full field envelope $\varPsi =\left|\varPsi \right|{e}^{i\varphi }$ is given by $\boldsymbol{\mathcal{J}}={k}_{0}^{-1}{\left|\varPsi \right|}^{2}\left({\nabla }_{\perp }\varphi -{k}_{0}{\beta }_{2}\frac{\partial \varphi }{\partial \tau }\vec{{\boldsymbol{\tau }}}\right)$ (Supplementary Note 5). It follows that when ${k}_{0}{\beta }_{2}=-1$, spatial diffraction and temporal dispersion are balanced, and the burst, composed of a set of STLG wavepackets, becomes a linear superposition of eigenmodes to wave equation^35^. This balance allows STOV pulses to hold their structure over long propagation distances. If the pulse burst propagates through air or dispersive medium without a matched group velocity dispersion (i.e., ${k}_{0}{\beta }_{2}\ne-\!\!1$), it will undergo a mode distortion^21,24^.

## Materials and methods

### Optical pulse burst and its spectral characteristics

Let’s start with a conventional 1D pulse burst characterized by invariant temporal separation, consistent across all pulses^8,9^. Neglecting spatial effects and using the slowly varying envelope approximation, an optical burst comprising *N* pulses with electric fields can be described as follows:

1 $${E}_{n}\left(t\right)={c}_{n}(t)\exp [i{\phi }_{n}\left(t\right)]$$

where ${c}_{n}(t)$ and ${\phi }_{n}\left(t\right)$ is the amplitude and phase of the *n*th envelop. The pulse burst is defined in the time domain as

2 $$E\left(t\right)=\mathop{\sum}\limits_{n=1}^{N}{E}_{n}\left(t-n\Delta \tau \right)$$

where $\Delta \tau$ being the intraburst temporal pitch. In our analysis, assuming all sub pulses are identical in amplitude, i.e., ${E}_{n}={E}_{p}$ for $n=\mathrm{1,2},\ldots ,N$. The spectral representation of Eq. (2) is expressed in the frequency domain as

3 $$\begin{array}{c}\widetilde{E}\left(\omega \right)\end{array}=\mathop{\sum}\limits_{n=1}^{N}{\widetilde{E}}_{n}\left(\omega \right)\exp \left({in}\Delta \tau \omega \right)$$

4 $$={\widetilde{E}}_{p}\left(\omega \right)\cdot \frac{1+{e}^{-{iN}\Delta \tau \omega }}{1+{e}^{-i\Delta \tau \omega }}$$

Equations (3) and (4) give the spectral representations of conventional pulse bursts. The last term in Eq. (4) shows the spectral interference factor as displayed in Fig. 1c, for *N* = 1, Eq. (4) reduces to a single pulse spectrum. We plot the formation of spectral modes of single pulse, *N* = 4 Gaussian pulse bursts with/without chirps in the Supplementary Note 10.

### Spatiotemporal wavepackets bursts and spatiotemporal multiplexing holography

To assign specific spatiotemporal coupling wavepackets to the comb teeth of a pulse burst, from Eq. (3), the equivalent spatial-spectral representation of a spatiotemporal vortex burst can be expressed as

5 $$E\left(\xi ,\Omega \right)=\sum _{p,{\mathcal{l}}}{c}_{p,{\mathcal{l}}}{{LG}}_{p}^{{\mathcal{l}}}(\xi ,\Omega )\exp {\rm{}}(i\Omega /{f}_{p,{\mathcal{l}}})$$

where $\Omega ={\omega -\omega }_{0}$ is the reduced angular frequency and ${{LG}}_{p}^{{\mathcal{l}}}(\xi ,\Omega )$ denotes Laguerre-Gaussian modes with the dual DoFs of the radial index *p* and azimuthal index *ℓ*^35^. ${c}_{p,{\mathcal{l}}}$ can be identical or different and determines the weights of each comb teeth and all comb teeth carry the identical frequency $\Omega ={\omega -\omega }_{0}$. The last term $\exp {\rm{}}(i\Omega /{f}_{p,{\mathcal{l}}})$ denotes the intraburst phase slip, which determinates the intraburst repetition rates. Note that such 2D spatial-spectral representation contains amplitude and phase information. As such, neglecting either the amplitude or the phase information results in strong crosstalks between each mode. Specifically, we adopt the following phase-only hologram to implement a complex-amplitude modulation^35,61^:

6 $${\psi }_{{\rm{SLM}}}\left(\xi ,\Omega \right)=\mathrm{mod}\left\{{\psi }_{E}+\pi {{\rm{sinc}}}^{-1}\left|E\right|+g\xi {{\rm{sinc}}}^{-1}[1-\left|E\right|]+\beta {\Omega }^{2},2\pi \right\}$$

where ${\psi }_{E}$ stands for the phase of $E\left(\xi ,\Omega \right)$ and $g$ denotes the frequency of a linear phase ramp, the depth of which is contingent upon the modulus of $E\left(\xi ,\Omega \right)$. The coherent superposition of LG modes of Eq. (5) generates a spatiotemporal multiplexing hologram [Eq. (6)], each mode carrying its own spatiotemporal features is, in principle, distinguishable from the others in the time domain. As such, after a space-time Fourier transformation, the resultant field demultiplexed along time dimension forming a pulse burst of spatiotemporal vortex pulses (see Supplementary Note 1):

7 $$E\left({r}_{x-\tau },{\theta }_{x-\tau }\right)=\sum _{p,{\mathcal{l}}}{c}_{p,{\mathcal{l}}}{{LG}}_{p}^{{\mathcal{l}}}({r}_{p,{\mathcal{l}}},{\theta }_{p,{\mathcal{l}}})$$

where ${r}_{p,{\mathcal{l}}}=\sqrt{{(\tau -{t}_{p,{\mathcal{l}}})}^{2}+{\beta }^{2}{x}^{2}}$, ${\theta }_{p,{\mathcal{l}}}={\tan }^{-1}[\beta x/(\tau -{t}_{p,{\mathcal{l}}})]$ and ${t}_{p,{\mathcal{l}}}=1/{f}_{p,{\mathcal{l}}}$. A spatiotemporal vortex burst consisting of isolated LG orthogonal basis is a solution to the scalar wave equation, which performs self-similar evolutions and remains pure vortex modes under dispersion matching conidiation to avoid intermodal crosstalks^35^.

## Supplementary information

Supplementary Information for Ultrafast bursts of tailored spatiotemporal vortex pulses

Supplementary Movie 1

## Data Availability

All other data are available in the article and its supplementary files or from the corresponding authors upon request.
