# Supplementary material for: Ultrafast bursts of tailored spatiotemporal vortex pulses
Source: Light Sci Appl. 2025 Oct 10;14:361. doi: 10.1038/s41377-025-02062-y (PMC12514292; doi:10.1038/s41377-025-02062-y)
Supplement: Supplementary file 1 — Supplementary Information for Ultrafast bursts of tailored spatiotemporal vortex pulses [file 41377_2025_2062_MOESM1_ESM.docx]

**Supplementary Information for**

**Ultrafast bursts of tailored spatiotemporal vortex pulses**

Xin Liu^1,2,3^, Chunhao Liang^2,3^, Qian Cao^1,4^, Yangjian Cai^2,3,*^, Qiwen Zhan^1,4,5,6,*^

^1^ School of Optical-Electrical and Computer Engineering, University of Shanghai for Science and Technology, Shanghai 200093, China.

^2^ Shandong Provincial Engineering and Technical Center of Light Manipulations and Shandong Provincial Key Laboratory of Optics and Photonic Device, School of Physics and Electronics, Shandong Normal University, Jinan 250014, China.

^3^ Collaborative Innovation Center of Light Manipulations and Applications, Shandong Normal University, Jinan 250358, China.

^4^ Zhejiang Key Laboratory of 3D Micro/Nano Fabrication and Characterization, Department of Electronic and Information Engineering, School of Engineering, Westlake University, Hangzhou, Zhejiang 310030, China.

^5^ Zhangjiang Laboratory, 100 Haike Road, Shanghai 201204, China.

^6^ International Institute for Sustainability with Knotted Chiral Meta Matter (WPI-SKCM2), Hiroshima University, Higashihiroshima, Hiroshima 739-8526, Japan.

^*^ Corresponding authors: yangjiancai@sdnu.edu.cn; qwzhan@usst.edu.cn.

**Supplementary Note 1: Experimental principle and apparatus for the generation and measurement of spatiotemporal vortex bursts**

A spatiotemporal multiplexing hologram, described by Eq. (5) in the main text, comprises four parts: (1) a composite phase of Eq. (5), (2) a compensation phase, (3) a spatial ramp phase whose depth modulated by the amplitude of Eq. (5), and (4) a group delay dispersion phase, as shown in Fig. S1. All components are superposed to synthesize a phase-only hologram to enable complex-amplitude modulation, as shown in Fig. S1. For the experimental setup, a chirped femtosecond pulse with a central wavelength of 1,030 nm and a bandwidth of ~20 nm is divided into two beams by a polarization beam splitter (PBS). One of these beams enters a folded 2D pulse shaper, comprising a grating (1,200 lines mm^-1^), cylindrical lens (CL with a focal length of 10cm), and reflective spatial light modulator (SLM, Holoeye GAEA-2, 3840×2160 pixels, 3.74 μm pixel size), loaded with a digital hologram as displayed in Fig. S1. The modulated pulses emitted from the grating spatially diffracted in distinct angles. We filtered out undesired lights using spatial blockers and allowing only the on-axial zeroth-order component to pass through^[1,2,3]^. The target spatiotemporal vortex burst is finally synthesized on the camera plane. Another pulsed beam passes through a pulse compressor and a time delay line and then is dechirped to an FTL pulse (~122 fs very close to the theoretical FTL 120 fs). After passing through a half-wave plate (HWP), it then interferes with the targeted pulse burst at a small angle through a BS. By scanning the time delay line, the 3D spatiotemporal characterization of the entire spatiotemporal vortex burst can be achieved from the delay-dependent interfered fringes^[4,5]^, as shown in Supplementary Note 4.


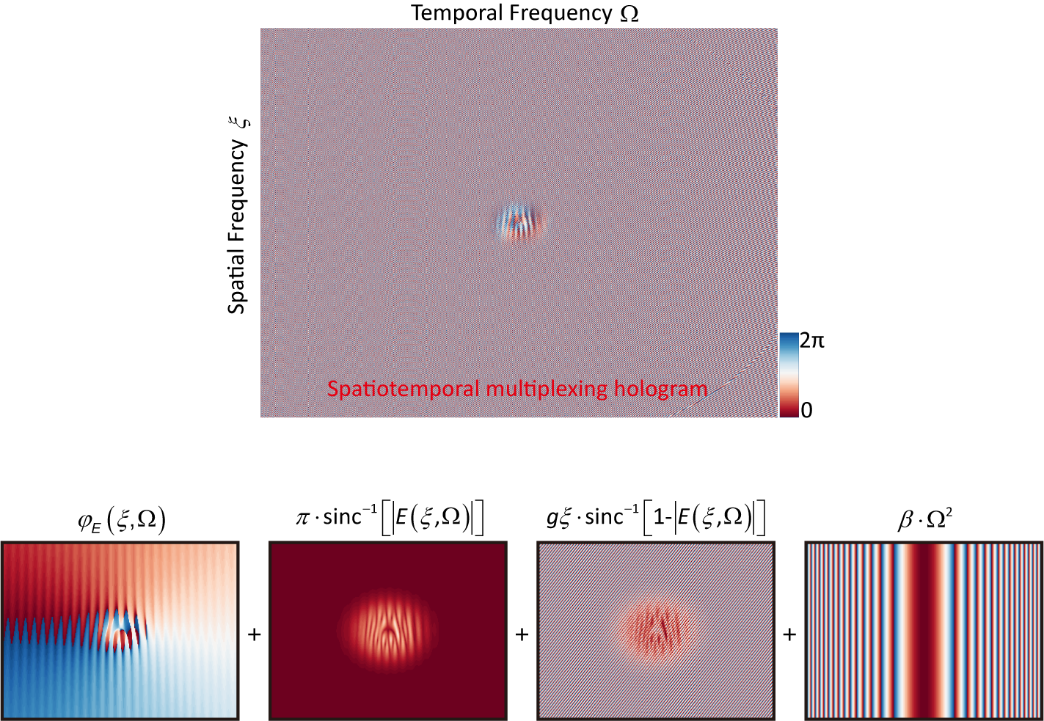


**Fig. S1 | Principle for spatiotemporal multiplexing hologram design.**

**Supplementary Note 2: Effects of the imbalance of spatial diffraction and temporal dispersion on the modal purity of the generated spatiotemporal vortex bursts**

In our experimental setup, the targeted spatiotemporal vortex bursts are synthesized at a specific plane $z=L$ after a distance behind a grating. Therefore, the spatiotemporal wavepackets have an additional diffractive phase $e^{{ik_{0}\xi^{2}}/{2L}}$ in their spatial components induced by the free-space propagation. To generate the space-time symmetric donut-shaped STOVs in this case, we must manage the dispersive phase $e^{iGDD\cdot\Omega^{2}}$ of the pulse to balance this diffractive phase term^[3]^. Fig. S2 shows the numerical characterizations of the GDD phase on the T-OAM modal purity of the generated spatiotemporal vortex bursts.


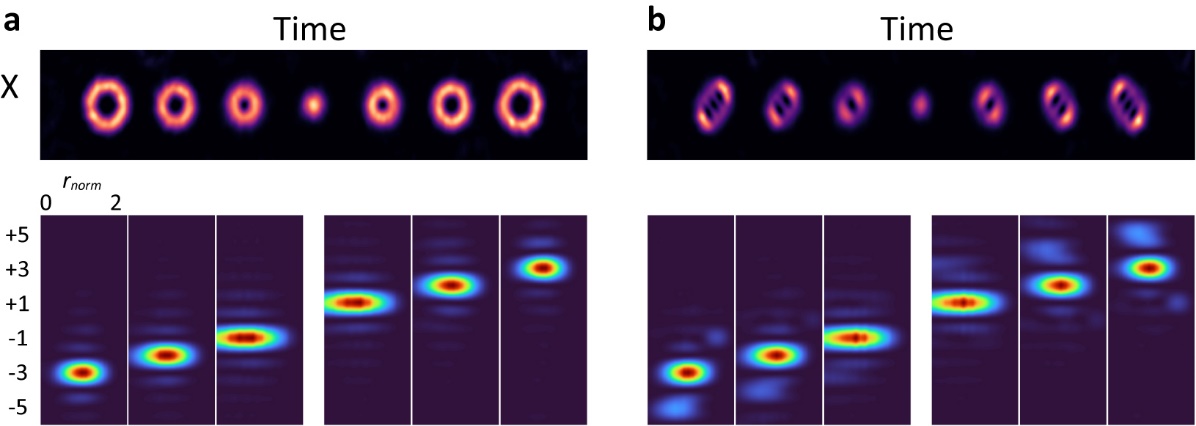


**Fig. S2 | Numerical analysis to characterize the impact of GDD on the modal purity of the produced spatiotemporal vortex bursts.** **a,** With a balanced $\beta={k_{0}}/{2L}=3952 \mathrm{fs}^{2}$. The T-OAM per photon in this case are calculated to be $-2.8229\hbar$, $-1.6510\hbar$, $-1.0172\hbar$, $-0.0296\hbar$, $0.9082\hbar$, $2.1578\hbar$, $+2.8689\hbar$. **b,** Without GDD. The T-OAM per photon in this case are calculated to be $-5.3174\hbar$, $-3.5024\hbar$, $-2.7273\hbar$, $-0.4860\hbar$, $+2.9197\hbar$, $+4.3192\hbar$, $+5.2724\hbar$.


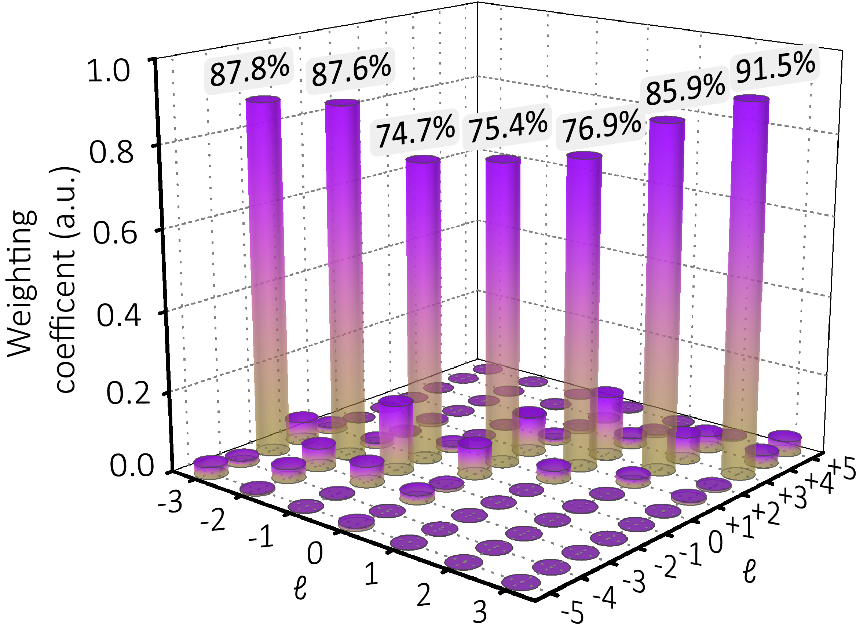


**Fig. S3 | Modal weight analysis corresponding to figure 3 in the manuscript, as outlined in Supplementary Note 8. The inserts denote the power distribution located at the desired mode indices.**

**Supplementary Note 3: Transverse OAM spectrum power analysis**

To estimate how much time-varying T-OAM spectrum power is located at the targeted mode indices, we analyzed the time-varying spatiotemporal vortices at their local space-time coordinates by utilizing the method adopted in the reference^[6]^. Unlike conventional L-OAM beams, the space-time coordinates of each spatiotemporal vortex comb teeth are varying inside the spatiotemporal vortex burst. As such, we first normalize the measured coordinates $(X,T)$ to the local spatiotemporal coordinates $(r_{\mathrm{norm}},\theta_{\mathrm{norm}})$ and $(\tau_{\mathrm{norm}},x_{\mathrm{norm}})$, where $r_{\mathrm{norm}}=\sqrt{{{(X-x_{0})}^{2}}/{w_{x}^{2}}+{{(T-t_{0})}^{2}}/{w_{t}^{2}}}$ and $\theta_{\mathrm{norm}}=\tan^{-1} \frac{{(X-x_{0})}/{w_{x}}}{{(T-t_{0})}/{w_{t}}}$. $(x_{0},t_{0})$ are the positions of the spatiotemporal phase singularity (or the midpoint of all multiple separate singularities positions) of each comb teeth inside the burst. We designate points $(x_{0},t_{0})$ as the origins and then proceed to draw cross lines along the time and space directions from these origins, resulting in two pairs of intensity peaks respectively. We then define the normalized widths $w_{t}$ and $w_{x}$ to be one-half of the peak-to-peak separations along the time and space directions, respectively. Finally, we can obtain the complex amplitude of these vortex-comb teeth $U(r_{\mathrm{norm}},\theta_{\mathrm{norm}})$ defined in their local polar coordinates. Hence, the T-OAM spectrum power wights can be calculated by

$c\left( r_{\mathrm{norm}},\mathcal{l} \right)=\left| \int_{0}^{2\pi} U(r_{\mathrm{norm}},\theta_{\mathrm{norm}})e^{-i\mathcal{l}\theta_{\mathrm{norm}}}d\theta_{\mathrm{norm}} \right|^{2}.$

In our work, we obtained Fig. 2c and Fig. S2 by scanning *ℓ* from -6 to +6 with an interval of 0.1.

**Supplementary Note 4: Measured interfered fringe patterns of generated bursts at some representative temporal slices**


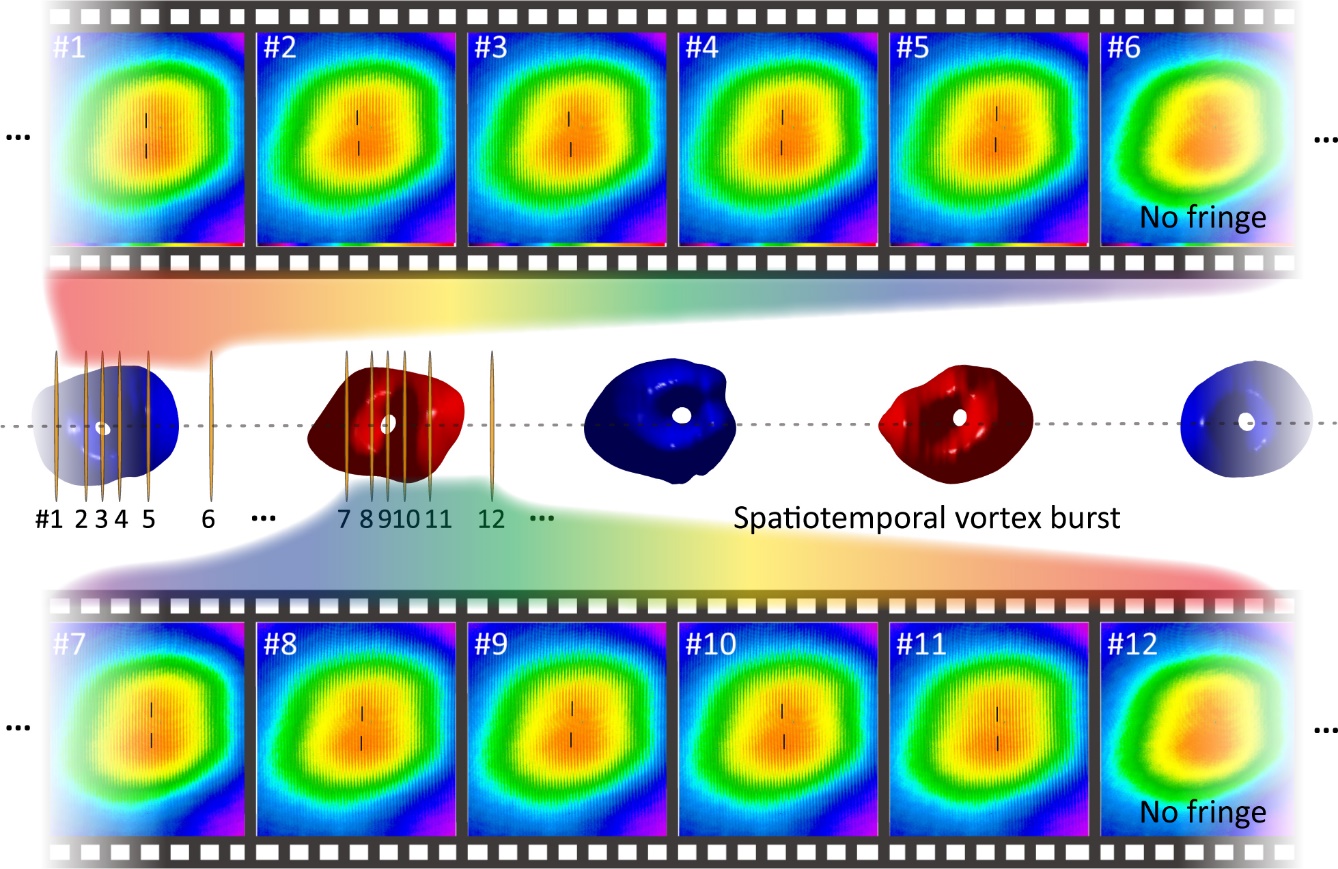


**Fig. S4 | Measured interfered fringe patterns of generated spatiotemporal vortex burst at some representative temporal slices by scanning time delays.**

**Supplementary Note 5:** **Energy density flux and mean T-OAM per photon calculation**

According to the electromagnetic theory of light and Maxwell's equations, the propagation of light pulses, represented by the electric field $E(x,y,z,t)$, in an isotropic, transparent, and linear medium can be described by the following Helmholtz equation

$\left( \frac{\partial^{2}}{\partial x^{2}}+\frac{\partial^{2}}{\partial y^{2}}+\frac{\partial^{2}}{\partial z^{2}} \right)E-\frac{n^{2}\left( \omega\right)}{c^{2}}\frac{\partial^{2}E}{\partial t^{2}}=0,$ (S1)

where, $c$ and $n\left( \omega\right)$ are the light speed in vacuum and the refractive index of medium, respectively. Considering the operator ${\partial\tilde{E}}/{\partial t=-i\omega_{0}\tilde{E}}$ in Fourier frequency domain of time, Eq. (1) can be rewritten as

$\left( \frac{\partial^{2}}{\partial x^{2}}+\frac{\partial^{2}}{\partial y^{2}}+\frac{\partial^{2}}{\partial z^{2}} \right)\tilde{E}+k^{2}\left( \omega\right)\tilde{E}=0.$ (S2)

Spatial and temporal Slow-Varying Envelope Approximations: (1) the distance traveled by light within the temporal duration of the pulse is much smaller than the wavelength, i.e., $\left| {\partial^{2}E}/{\partial z^{2}} \right|\ll\left| k_{0}{\partial E}/{\partial z} \right|\ll\left| k_{0}^{2}E \right|$; and (2) the pulse envelope width is much larger than the carrier wave period, i.e., $\left| {\partial^{2}E}/{\partial t^{2}} \right|\ll\left| \omega_{0}{\partial E}/{\partial t} \right|\ll\left| \omega_{0}^{2}E \right|$, make the electric field $E(x,y,z,t)$ reduced to be

$E\left( x,y,z,t \right)=\Psi\left( x,y,z,t \right)\exp(-i\omega_{0}t+ik_{0}z),$ (S3)

where $\Psi\left( x,y,z,t \right)$ is a slow-varying envelope. Similarly, Eq. (3) has a Fourier form as $\tilde{E}\left( x,y,z,\omega\right)=\tilde{\Psi}\left( x,y,z,\Omega\right)\exp(ik_{0}z)$, where $\Omega=\omega-\omega_{0}$. Then, Eq. (S2) can be reduced to

$\left( \frac{\partial^{2}}{\partial x^{2}}+\frac{\partial^{2}}{\partial y^{2}} \right)\tilde{\Psi}+2ik_{0}\frac{\partial\tilde{\Psi}}{\partial z}+\left[ k^{2}\left( \omega\right)-k_{0}^{2} \right]\tilde{\Psi}=0,$ (S4)

where Taylor expands $k^{2}\left( \omega\right)-k_{0}^{2}\approx2k_{0}\left[ k\left( \omega\right)-k_{0} \right]=2k_{0}k^{\left( 1 \right)}\left( \omega_{0} \right)\Omega+k_{0}k^{\left( 2 \right)}(\omega_{0})\Omega^{2}$. Also consider variable substitution $\varsigma=z$ and $\tau=t-k^{\left( 1 \right)}\left( \omega_{0} \right)z$, Eq. (S4) can be written in the time domain as

$\left( \frac{\partial^{2}}{\partial x^{2}}+\frac{\partial^{2}}{\partial y^{2}}-k_{0}\beta_{2}\frac{\partial^{2}}{\partial\tau^{2}} \right)\Psi(x,y,z,\tau)+2ik_{0}\frac{\partial\tilde{\Psi}}{\partial\varsigma}=0,$ (S6)

where $\beta_{2}$ is the group velocity dispersion (GVD) coefficient of the dispersive medium. Following Ref.[7,8,9], the total energy $\int_{V} \left| \Psi\left( x,y,\varsigma;\tau\right) \right|^{2}dxdyd\tau$ satisfies an energy conservation equation, which can be derived by multiplying Eq. (S6) by a complex conjugate of the wavepacket, and is given by (without integral)

$\frac{\partial\left| \Psi\right|^{2}}{\partial\varsigma}=-\frac{i}{2k_{0}}\left[ \frac{\partial}{\partial x}\left( \Psi\frac{\partial\Psi^{*}}{\partial x}-\Psi^{*}\frac{\partial\Psi}{\partial x} \right)+\frac{\partial}{\partial y}\left( \Psi\frac{\partial\Psi^{*}}{\partial y}-\Psi^{*}\frac{\partial\Psi}{\partial y} \right) \right]-i\frac{\beta_{2}}{2}\frac{\partial}{\partial\tau}\left( \Psi^{*}\frac{\partial\Psi}{\partial\tau}-\Psi\frac{\partial\Psi^{*}}{\partial t} \right).$ (S7)

Comparing Eq. (S7) with the definition of the divergence operator, Eq. (S7) yields

$\frac{\partial\left| \Psi\right|^{2}}{\partial\varsigma}=-\text{div}\mathbf{J}=-\nabla_{\perp}\cdot\boldsymbol{J}_{\perp}-\frac{\partial\boldsymbol{J}_{\tau}}{\partial\tau},$ (S9)

with

$\boldsymbol{J}_{\perp}=\frac{i}{2k_{0}}\left( \Psi^{*}\nabla_{\perp}\Psi-\Psi\nabla_{\perp}\Psi^{*} \right),$ (S10)

$\boldsymbol{J}_{\tau}=i\frac{\beta_{2}}{2}\left( \Psi^{*}\frac{\partial\Psi}{\partial\tau}-\Psi\frac{\partial\Psi^{*}}{\partial\tau} \right)\vec{\boldsymbol{\tau}}.$ (S11)

where $\nabla_{\perp}=\frac{\partial}{\partial x}\vec{\boldsymbol{x}}+\frac{\partial}{\partial y\vec{\boldsymbol{y}}}$. The cross product of the energy density flux $\boldsymbol{J}$ with position vector $\vec{\boldsymbol{r}}=x\vec{\boldsymbol{x}}+y\vec{\boldsymbol{y}}+\tau\vec{\boldsymbol{\tau}}$ gives an OAM density flux $\vec{\boldsymbol{L}}={\vec{\boldsymbol{L}}}_{x}+{\vec{\boldsymbol{L}}}_{y}+{\vec{\boldsymbol{L}}}_{\tau}$, where ${\vec{\boldsymbol{L}}}_{y}$ is the T-OAM along the y direction and given by

$\hat{L}_{y}=-i\left( \tau\frac{\partial}{\partial x}+xk_{0}\beta_{2}\frac{\partial}{\partial\tau} \right).$ (S11)

It is worth noting that each vortex-comb teeth within the spatiotemporal vortex burst are independent, unlike conventional ones embedded within a whole packet as discussed in^[17,18]^. Therefore, we can calculate their mean T-OAM per photon in their normalized spatiotemporal coordinate respectively [Fig. S5], with the following equation^[4,6]^:

$\left\langle L_{y} \right\rangle=\hbar\frac{\left\langle\Psi|\hat{L}_{y}|\Psi\right\rangle}{\left\langle\Psi|\Psi\right\rangle}=\hbar\frac{\int\Psi^{*}|\hat{L}_{y}\Psi d\tau_{\mathrm{norm}}dx_{\mathrm{norm}}}{\int\Psi^{*}\Psi{d\tau}_{\mathrm{norm}}dx_{\mathrm{norm}}},$ (S11)

where $(\tau_{\mathrm{norm}},x_{\mathrm{norm}})$ is the spatiotemporal normalized coordinate, as described in Supplementary Note 3. The experimentally calculated T-OAM/photon values exhibits somewhat fluctuations with the theoretical ones because the asymmetric intensity distributions and imperfect helical phases^[4]^.


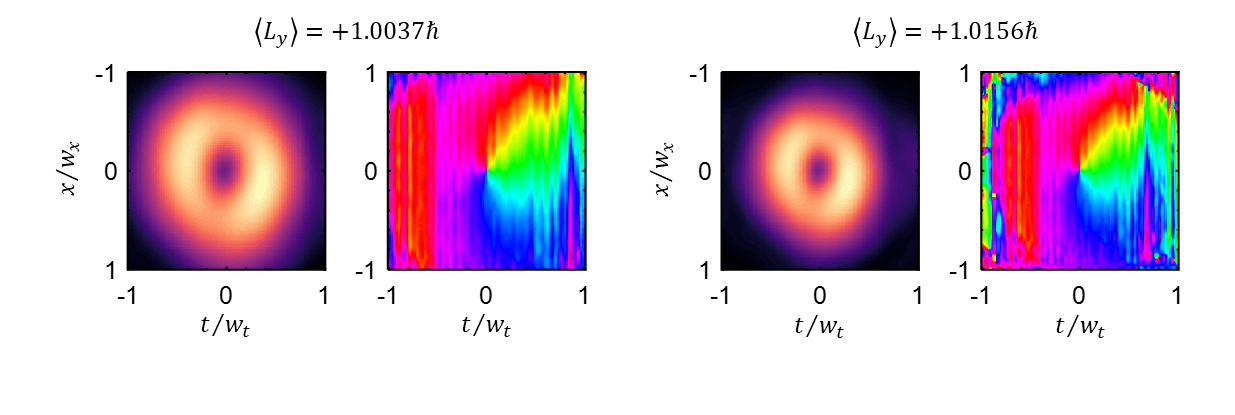


**Fig. S5 | Calculated mean T-OAM value of an STOV comb teeth in the local spatiotemporal coordinate. Scaling coordinates does not affect the calculated mean T-OAM, which remains essentially unchanged (the subtle change comes from the residual background intensity).**

On the other hand, the spatiotemporal wavepacket at a distance 𝐿 from the pulse shaper exhibits a profile as follow

$$\Psi\left( x,y,L,\tau\right)=\frac{\exp\left[ ik_{0}L+\frac{ik_{0}\left( x^{2}+y^{2} \right)}{2L} \right]}{2\pi}\times\iiint\Psi\left( x_{0},y_{0},\Omega\right)$$

$\exp\left( ik_{0}\frac{x_{0}^{2}+y_{0}^{2}}{2L}+i\mathrm{GDD}\cdot\Omega^{2} \right)\exp\left( -ik_{0}\frac{xx_{0}+yy_{0}}{L}-i\Omega\tau\right)d\Omega dx_{0}dy_{0}$ (S12)

and a corresponding angular spectrum form

$\Psi\left( x,y,L,\tau\right)=\frac{e^{ik_{0}L}}{8\pi^{3}}\iiint\tilde{\Psi}\left( k_{x},k_{y},\Omega\right)H\left( k_{x},k_{y},\Omega\right)\exp\left( -ik_{x}x-ik_{y}y-i\Omega\tau\right)d\Omega dk_{x}dk_{y}.$(S13)

where $H\left( k_{x},k_{y},\Omega\right)=\exp\left[ -i{\left( k_{x}^{2}+k_{y}^{2} \right)L}/{2k_{0}}+{i\beta_{2}\Omega^{2}L}/2 \right]$. Eq. (S12) and Eq. (S13) are completely equivalent, except that the latter is expressed in momentum space. In our experiment, targeted spatiotemporal vortex bursts were synthesized at a plane located a distance *L* behind the pulse shaper. By manipulating the GDD phase applied to the SLM, we balanced spatial diffraction and temporal dispersion, as described by Eq. (S12). The positive GDD magnitude was set to ${k_{0}a^{2}}/{2L}$, where $a$ represents the ratio of the spatial to spectral width of the pulse^[3]^. Under this condition, the spatiotemporal optical vortices are spatiotemporally symmetry and have a perfect helical phase structure. In this case, from Eq. (S13), we obtain $\beta_{2}={-b^{2}}/{k_{0}}$, where $b$ is the ratio of the spatial-frequency to spectral width of the pulse in momentum space. According to Eq. (S11), we plot the energy density flux in Fig. 3c with $\beta_{2}={-1}/{k_{0}}$ and in Fig. S6 and the dominant linear momentum pointing in the $z$ or $t$ direction is removed. According to Eq. (S11), we can calculate the average T-OAM per photon values in Fig. 3c and Fig. S6 in the local coordinates (Supplementary Note 3) with the ratio of the *x*-axis to the *t*-axis set to 1.


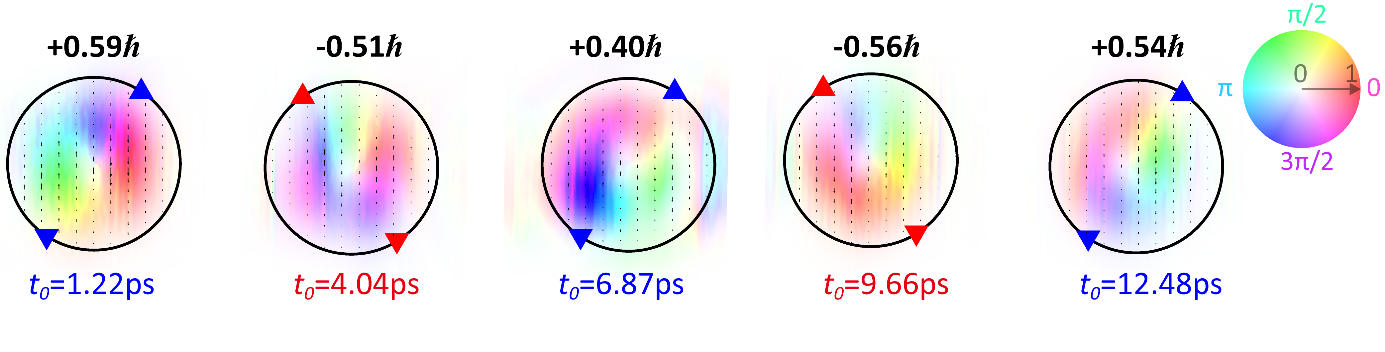


**Fig. S6 | Complex field and energy density flux (calculated with** $\boldsymbol{\beta}_{\boldsymbol{2}}\boldsymbol{=0}$**) of pulse burst in the space-time domain,** **the energy density flux of each teeth is restricted to x-axis. The values at the top denote the mean value of the T-OAM** $\left\langle\boldsymbol{L}_{\boldsymbol{y}} \right\rangle$ **per photon for each comb teeth and agree well with a half of topological charge. The saturation and hue denote amplitude and phase information respectively.**

**Supplementary Note 6: Optical analogy of Kármán Vortex Street in the linear space-time domain**

In fluid dynamics, a Kármán vortex street (KVS or von Kármán vortex street) is a recurring pattern of swirling vortices created by the nonlinear process of vortex shedding. This process leads to the unsteady separation of fluid flow around blunt bodies. Animation. S1 displays a typical animation of a two-dimensional KVS created by a cylindrical object (online available at <https://en.wikipedia.org/wiki/K%C3%A1rm%C3%A1n_vortex_street>) and a typical movie of a KVS created by an airfoil (online available at <https://www.youtube.com/watch?v=k9FPxuhFlTo>), exhibits a time-varying charily of T-OAMs. In addition, 2D KVS-like optical fields were reported early in^[10,11,12,13]^ and 3D recently reported in^[14,15]^. Here, the Animation. S1 and Fig. 3 of the main text are examples of KVSs with 2D structures. The 3D KVS optical fields can also be generated by the integration of our scheme and conformal mapping method^[15,16]^. Besides, the generalized KVSs (including high-order vortices and multiple rings structures, etc.) can also be further created, as shown in Fig. S4c and Fig. 4 of the main text.


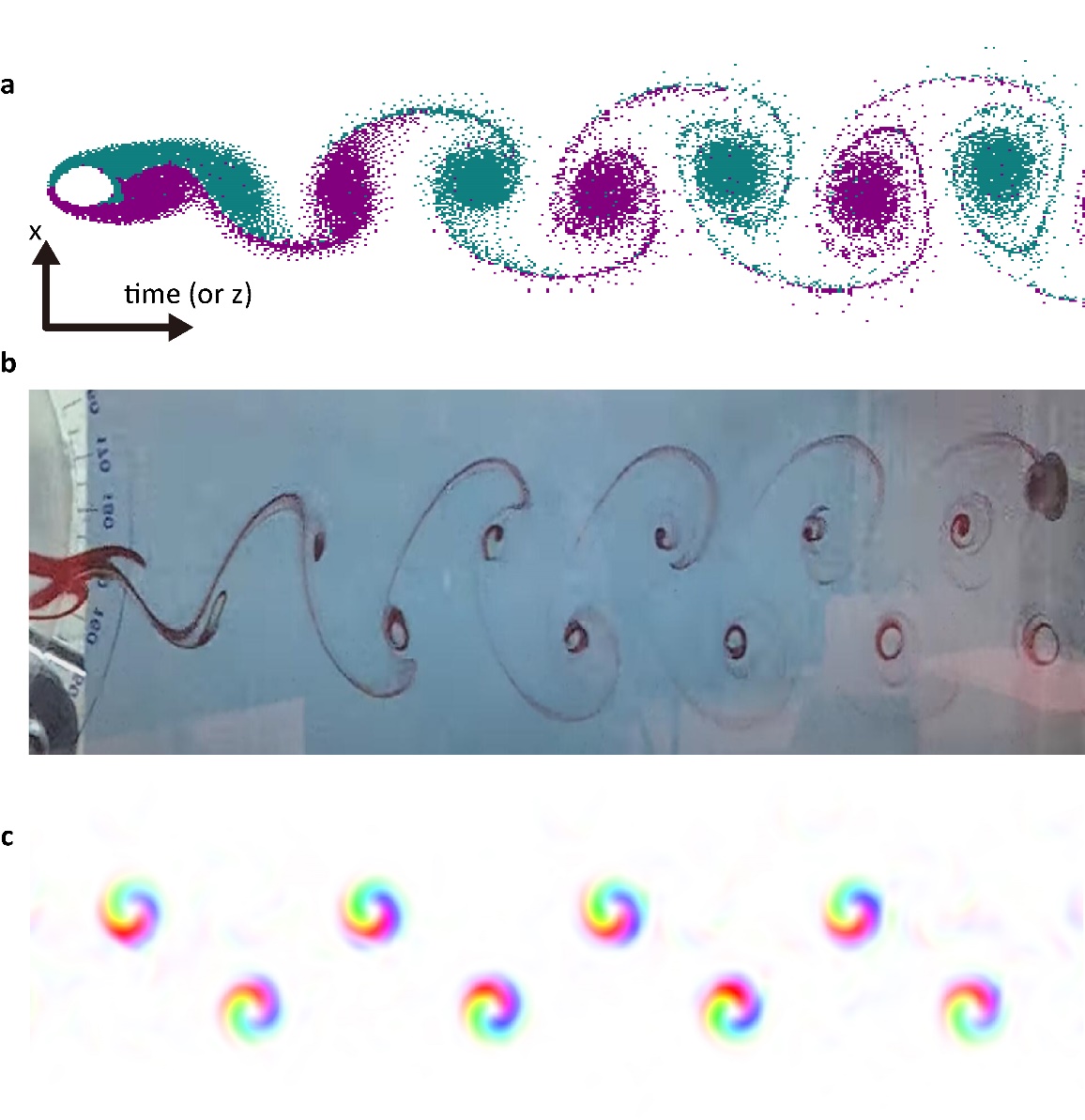


**Animation. S1 | Some Visualizations of 2D KVSs: a, created by a cylindrical (symmetric) object, in which the vortices with right and left handed charily were denoted by purple and cyan, respectively. b and c, created by an airfoil (asymmetric) and its optical analogies with our spatiotemporal vortex pulse bursts.** (**a,** [Animation online](https://en.wikipedia.org/wiki/K%C3%A1rm%C3%A1n_vortex_street); **b,** [Movie online](https://www.youtube.com/watch?v=k9FPxuhFlTo);)

**Supplementary Note 7: Spatiotemporal vortex bursts with controllable intraburst repetition rates and arrangements**

The time pitch of vortex comb teeth inside the spatiotemporal vortex bursts can be linearly regulated by adjusting the intraburst phase slips. For example, Fig. S7a shows a comparable result of Fig. 3 but with a time pitch of 1.6 ps. Fig. S7b presents the measured temporal separations as a function of their intraburst phase slips. The minimum time pitch is constrained by the spatiotemporal breadth of adjacent vortex comb teeth since their spatiotemporal coherence, as expounded upon in Supplementary Note 9 regarding spatiotemporal collisions. The spatiotemporal radius of vortex comb teeth follows $w_{0}\sqrt{2p+\left| \mathcal{l} \right|+1}$, where the beam waist $w_{0}$ is solely determined by the carried chirp of wavepacket^[3,4]^. Consequently, the time pitch within spatiotemporal vortex bursts can be further reduced by decreasing the chirp of the pulse^3^.

It should be noted that each vortex comb teeth exists independently rather than being embedded within a single wavepacket^9,10^. As such, for the intensities and phases of winding, two adjacent vortex comb teeth with the same *ℓ* are fundamentally distinct from the vortex comb teeth with 2*ℓ* within a spatiotemporal vortex burst, as plotted by the typical example in Fig. S7c. Additionally, since the individual comb teeth within a burst are isolated, they exhibit high modal purity, as illustrated in Fig. S7d.


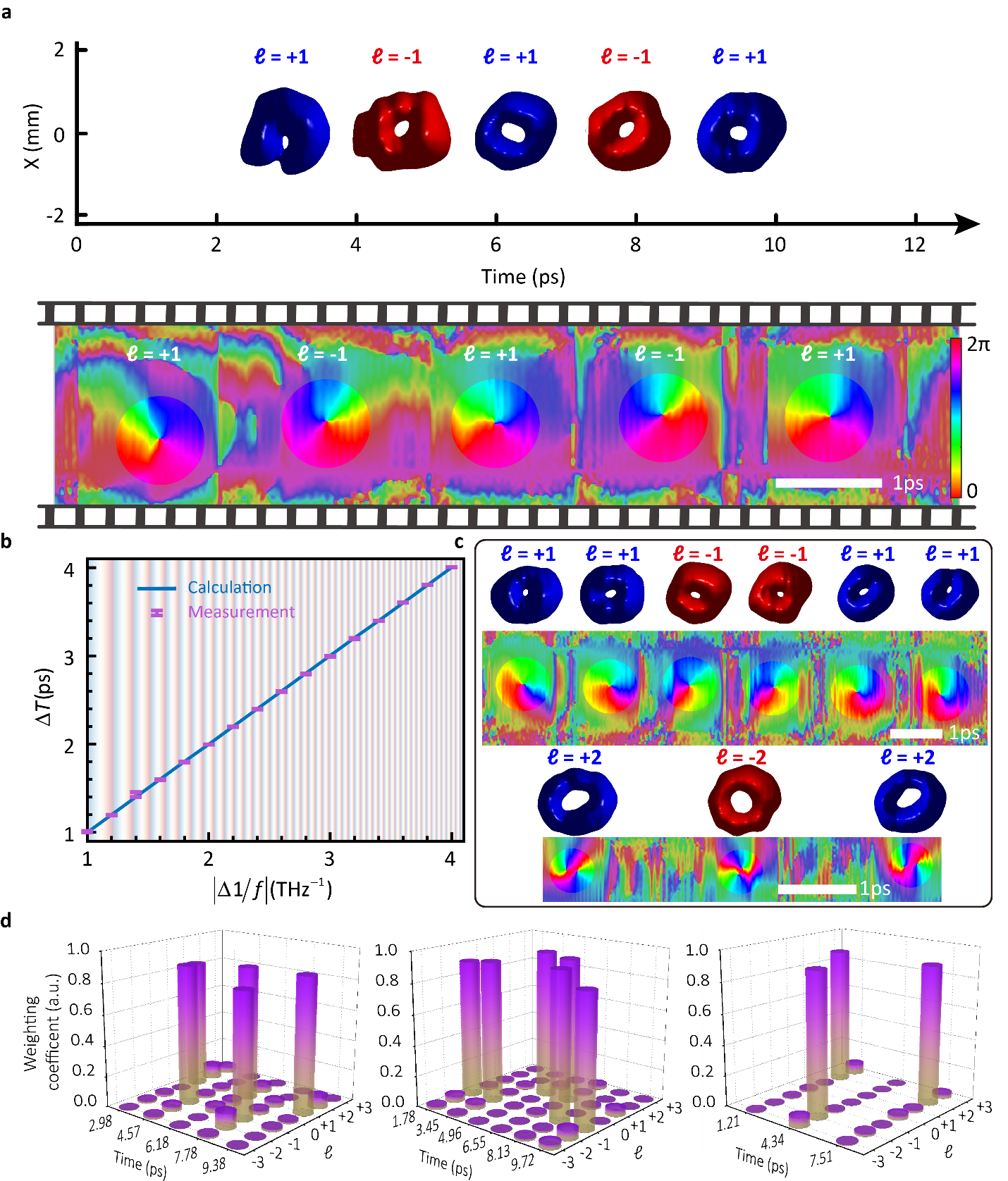


**Fig. S7 | Experimental demonstration of spatiotemporal vortex bursts with tunable temporal pitch and arrangements. a,** Measured iso-intensity profile and spatiotemporal phase distribution of Fig. 3 but with a smaller time pitch of 1.6 ps. **b,** Measured temporal separation curves as a function of the intraburst phase slips. **c,** Demonstration of spatiotemporal vortex bursts with self-defined time-varying orders. **d,** Calculated mode purity of **a** and **c** (Supplementary Note 8).

**Supplementary Note 8: Modal analysis of the generated spatiotemporal bursts**

To estimate modal purity of each comb teeth within the burst, we can perform the modal decomposition for each comb teeth by a set of LG basis in the normalized space-time coordinates:

$E\left( T,X \right)=\sum_{p=0}^{+\infty} \sum_{l=-\infty}^{+\infty} C_{p\mathcal{,l}}{LG}_{p}^{\mathcal{l}}\left( T,X \right),$ (S14)

the modal weight coefficient $\left| C_{p,l} \right|^{2}$ can be calculated from

$C_{p\mathcal{,l}}=\iint E(T,X)\left[ {LG}_{p}^{\mathcal{l}}\left( T,X \right) \right]^{*}dTdX,$ (S15)

where * denotes the complex conjugate. The power distribution located at desired radial $p$ and azimuthal $\mathcal{l}$ index is defined as ${\left| C_{p\mathcal{,l}} \right|^{2}}/{\sum_{p\mathcal{,l}} \left| C_{p\mathcal{,l}} \right|^{2}}$. From Eq. (S2), the wave equation admits a solution in the form of an LG wave function, given by^[3]^

$\mathcal{L}_{p}^{\mathcal{l}}\left( r,\theta,z \right)=\sqrt{\frac{2p!}{\pi\left( p+\left| \mathcal{l} \right| \right)!}}\frac{1}{w_{z}}\left( \frac{\sqrt{2}r}{w_{z}} \right)^{\left| \mathcal{l} \right|}L_{p}^{\left| \mathcal{l} \right|}\left( \frac{2r^{2}}{w_{z}^{2}} \right)$

$\times exp\left[ -\frac{2r^{2}}{w_{z}^{2}}+i\left( \mathcal{l}\theta+\frac{k_{0}r^{2}}{2R_{z}}-(2p+\left| \mathcal{l} \right|+1)\tan^{-1} \frac{2z}{k_{0}w_{0}^{2}} \right) \right]$, (S16)

where $w_{z}=w_{0}\sqrt{1+{4z^{2}}/{k_{0}w_{0}^{4}}}$, $R_{z}=z+{k_{0}w_{0}^{4}}/{4z}$, $w_{0}$ is the beam with of a fundamental Gaussian function. $r=\sqrt{x^{2}+t^{2}}$ and $\theta=\tan^{-1} x/t$ is the local polar coordinate. Based on Eq. (S15), the spectrum power located at desired radial *p* and azimuthal $\mathcal{l}$ index is calculated as $C_{p\mathcal{,l}}=\iint\mathcal{L}_{p}^{\mathcal{l}}\left( r,\theta,0 \right)\left[ \mathcal{L}_{p}^{\mathcal{l}}\left( r,\theta,\Delta z \right) \right]^{*}dtdx$. Since the radial quantum number governs the beam’s intrinsic hyperbolic momentum^[19]^, the radial mode purity is highly sensitive to the LG mode’s radial component. Figure S8 illustrates that as the radial quantum number $p$ of the LG mode increases, the degradation of mode purity due to intensity and phase mismatches becomes more pronounced. However, in experiments, imperfections in the generated STLG wavepacket caused by noise and optical elements are unavoidable. These subtle mismatches in intensity and phase between the generated and ideal STLG wavepackets for large radial quantum numbers are the primary factor contributing to the lower mode purity of high-order radial index STLG wavepackets compared to their low-order counterparts.


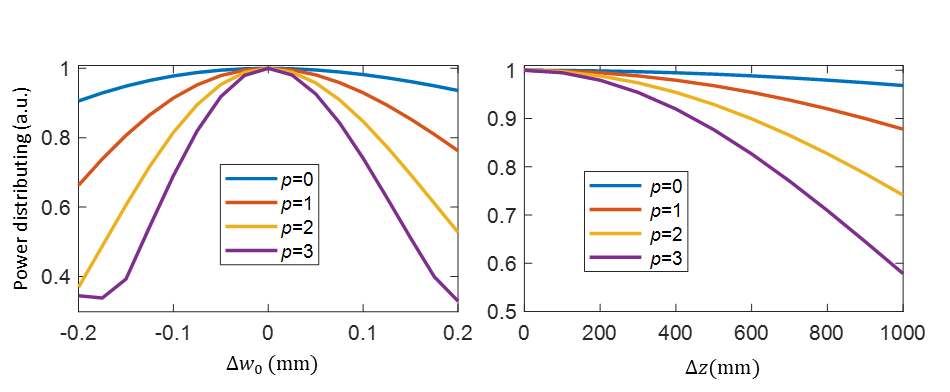


**Fig. S8 | Power distribution at the desired radial and azimuthal indices of LG mode of** $\mathcal{l}$**=1** ${\left| \boldsymbol{C}_{\boldsymbol{p,}\mathcal{l}} \right|^{\boldsymbol{2}}}/{\sum_{\boldsymbol{p,}\mathcal{l}} \left| \boldsymbol{C}_{\boldsymbol{p,}\mathcal{l}} \right|^{\boldsymbol{2}}}$ **as a function of the mismatch in the radial width and distance the modes have propagated.**

**Supplementary Note 9: Spatiotemporal collision of STLG wavepackets within a spatiotemporal vortex burst**

By changing the intraburst phase slips, we achieve a spatiotemporal collision of STLG wavepackets within a spatiotemporal vortex burst. We first generate a spatiotemporal STLG wavepacket burst with time-varying radial and azimuthal quantum numbers. The sequence is set to *p*=3, *ℓ*=+4, *p*=3, *ℓ*=+4, *p*=1, *ℓ*=+2 and temporal pitch is set to 4 ps. Then we delay the first wavepacket in time up to 4 ps to collide with the second wavepacket in the space-time domain, forming a petal-like (has $p+1=4$ layers and $2\left| l \right|=8$ petals) interference pattern, as shown in Fig. S9.


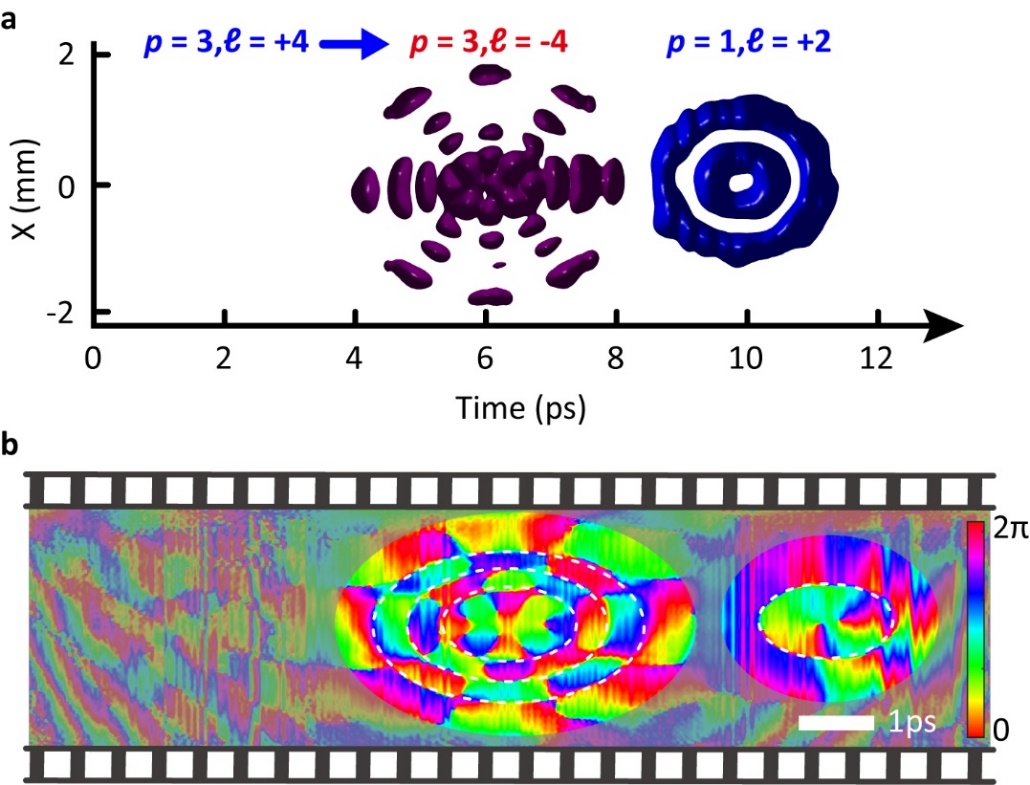


**Fig. S9 | Experimental demonstration of the spatiotemporal collision of two STLG wavepackets (*p*=3, *ℓ*=+4 at 2 ps and *p*=3, *ℓ* =-4 at 6 ps) within a spatiotemporal vortex burst consisting of a train of STLG wavepackets with time-varying *p* and *ℓ*. a,** Measured iso-intensity (at 5% of peak intensity) profile of the burst, which depicts two STLG wavepackets with *p*=3, *ℓ*=+4 and *p*=3, *ℓ*=-4 are collided at 6 ps, results in a distinctive petal-like spatiotemporal interference pattern. **b,** Spatiotemporal phase distribution.

**Supplementary Note 10: Spectral characteristics of 1D pulse bursts**

**
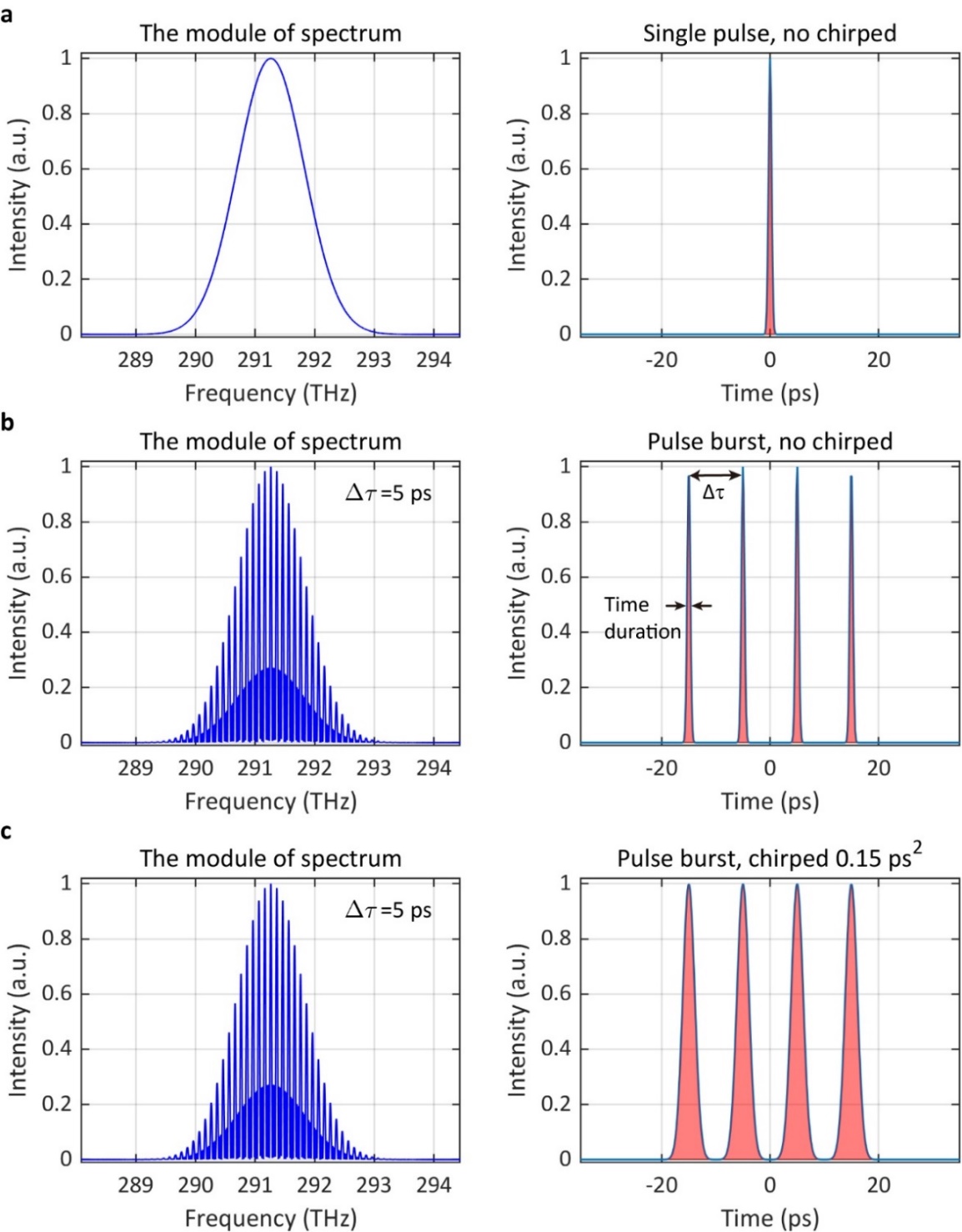
**

**Fig. S10 | Spectral representations and corresponding pulse bursts of conventional 1D temporal optical pulses. a,** For a single pulse without a chirp. **b,** For a pulse burst without a chirp. **c,** For a pulse burst with a linear chirp of 0.15 ps^2^.

**Supplementary Note 11: Numerical demonstration of a spatiotemporal wavepacket burst consisting of intricate spatiotemporal structure wavepackets**

**
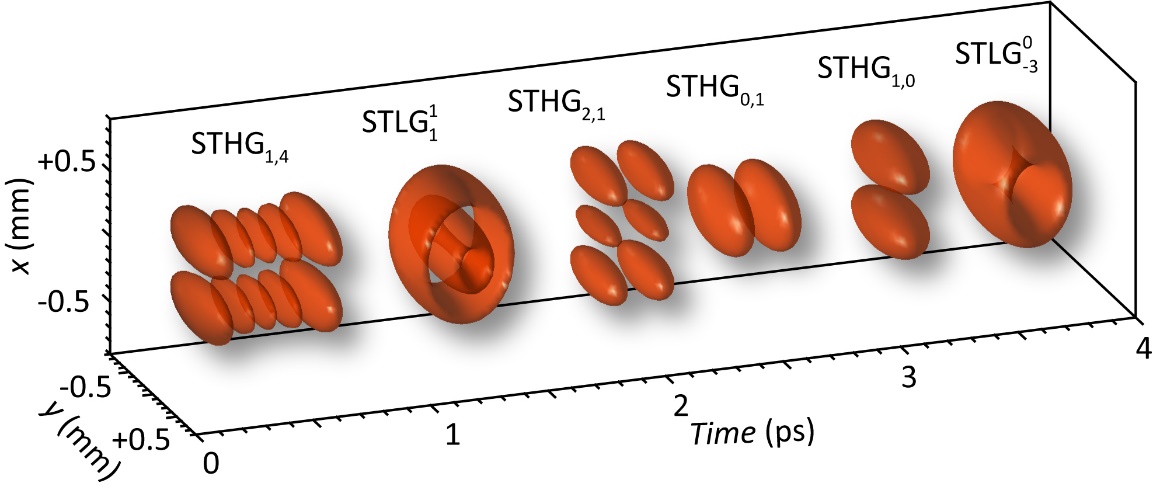
**

**Fig. S11 | Intricate tailored spatiotemporal wavepacket burst consisting of a set of STLG*^p^_l_* and spatiotemporal Hermite-Gaussian (STHG*_m,n_*) basis with various orders.** The isovalue is set to 2% of peak intensity.

**Supplementary Note 12: Analysis of the upper bound on the number of comb teeth in a burst**

The maximum number of comb teeth within a pulse burst is typically limited by the spatial-spectral resolution of the 2D pulse shaper. This resolution is influenced by several factors such as the SLM, gratings, cylindrical lenses, and the input spatial size and bandwidth. For a Gaussian pulsed beam of a $1/e$ radius $w_{i}$, the beam width of each frequency at spatial-spectral plane is $w_{freq}=\frac{2f\cos\theta_{i}}{k_{0}w_{i}\cos\theta_{d}}$, where $f$ is a focal length of the cylindrical lens, $\theta_{i}$ and $\theta_{d}$ is the incident angle and 1^st^ order diffractive angle on/from the grating, $k_{0}={2\pi}/{\lambda_{0}}$. Based on our experimental conditions, the input pulsed beam before the pulse shaper has parameters: $w_{i}=2$ mm; bandwidth $\Delta\lambda=20$ nm; center wavelength $\lambda_{0}=1030$ nm; $\theta_{i}=46^{\circ}$; $f=10$cm; grating with $N=1,200$ lines and SLM spatial resolution $\delta x=3.74$μm, the beam width of each frequency on SLM and the spectral sample rate by SLM can be calculated to be $2w_{freq}=26.56$ μm and $\left| \delta\omega\right|=4.76e^{-5}$ rad/fs/pixel, respectively. (i) The Nyquist-Shannon sampling theorem dictates each frequency component be sampled by, at least, two pixels on the SLM, that is ${2w}_{freq}\geq2\delta x$. This condition is easily satisfied in our experiment. (ii) Besides, the spectral interference factor of pulse bursts [as described by Eq. (4) in the main text] is a truncated cosine function about *N*, i.e. $\propto\cos{(N\Delta\tau\omega}/{2)}$, it has an eigen frequency $f_{N}={N\Delta\tau}/{4\pi}$, which is unrelated to the chirp phases. Based on Nyquist-Shannon sampling theorem, in order to generate high-fidelity pulse bursts, this frequency must satisfy ${2f}_{N}\leq\left| \delta\omega\right|^{-1}$, set a theoretical upper bound $N\leq{2\pi}/\left| \Delta\tau\cdot\delta\omega\right|$ on the pulse number of the generated bursts in our work. Furthermore, to minimize interference and crosstalk while maintaining high T-OAM purity, the time interval $\Delta\tau$ must exceed the full temporal width of the STOV pulse, given by ${2w}_{p,l}={2w}_{0}\sqrt{2p+\left| \mathcal{l} \right|+1}$, where $w_{0}$ is the temporal width of a fundamental Gaussian pulse. To produce high-quality STOV modes at specific teeth, the pulsed beam’s spatial-spectral bandwidths should entirely cover the spatiotemporal multiplexing hologram loaded on the SLM. Hence, the spatial-spectral bandwidths of input pulsed beam and the spatial resolution of the SLM jointly impose a constraint on the generation of maximum order STOV pulse ^[3]^.

**References:**

1. Cundiff S. T. & Weiner A. M. Optical arbitrary waveform generation. *Nat. Photonics* **4**, 760-766 (2010).
2. Cao, Q., Zhang, N., Chong, A. & Zhan, Q. Spatiotemporal Hologram. arXiv:2401.12642 (2024).
3. Liu, X., Cao, Q., Zhang, N., Chong, A., Cai, Y. & Zhan, Q. Spatiotemporal optical vortices with controllable radial and azimuthal quantum numbers. *Nat. Commun.* **15**, 5435 (2024).
4. Chong, A., Wan, C., Chen, J. & Zhan, Q. Generation of spatiotemporal optical vortices with controllable transverse orbital angular momentum. *Nat. Photonics* **14**, 350-354 (2020).
5. Li, H., Bazarov, I. V., Dunham, B. M. & Wise, F. W. Three-dimensional laser pulse intensity diagnostic for photoinjectors. *Phys. Rev. ST Accel. Beams* **14**, 112802 (2011).
6. Gui, G., Brooks, N. J., Kapteyn, H. C., Murnane, M. M. & Liao, C. T. Second-harmonic generation and the conservation of spatiotemporal orbital angular momentum of light. *Nat. Photonics* **15**, 608-613 (2021).
7. Jhajj, N. et al. Spatiotemporal optical vortices. *Phys. Rev. X* **6**, 031037 (2016).
8. Faccio, D. et al. Experimental energy-density flux characterization of ultrashort laser pulse filaments. *Opt. Express* **17**, 8193–8200 (2009).
9. Lotti, A., Couairon, A., Faccio, D. & Di Trapani, P. Energy-flux characterization of conical and space-time coupled wave packets. *Phys. Rev. A* **81**, 023810 (2010).
10. Scheuer, J. & Orenstein, M. Optical vortices crystals: Spontaneous generation in nonlinear semiconductor microcavities. *Science* **285**, 230-233 (1999).
11. Molina-Terriza, G., Petrov, D. V., Recolons, J. & Torner, L. Observation of optical vortex streets in walking second-harmonic generation. *Opt. Lett.* **27**, 625-627 (2002).
12. Molina-Terriza, G., Petrov, D. V. & Torner, L. Singular optics: Optical vortex streets. *Optics and Photonics News* **13**, 56-56 (2002).
13. Shen, Y., Wan, Z., Fu, X., Liu, Q. & Gong, M. Vortex lattices with transverse-mode-locking states switching in a large-aperture off axis-pumped solid-state laser. *JOSAB* **35**, 2940-2944 (2018).
14. Shen, Y., Papasimakis, N., & Zheludev, N. I. Nondiffracting Supertoroidal Pulses: Optical" Kármán Vortex Street". ArXiv:2204.05676 (2022).
15. Shen, Y., Papasimakis, N. & Zheludev, N.I. Nondiffracting supertoroidal pulses and optical “Kármán vortex streets”. *Nat. Commun.* **15**, 4863 (2024).
16. Wan, C., Cao, Q., Chen, J., Chong, A. & Zhan, Q. Toroidal vortices of light. *Nat. Photonics***16**, 519-522 (2022).
17. Huang, S., Zhang, N., Lu, X., Liu, J. & Yao, J. Spatiotemporal vortex strings of light. ArXiv:2305.08407 (2023).
18. Wan, C., Chen, J., Chong, A. & Zhan, Q. Generation of ultrafast spatiotemporal wave packet embedded with time-varying orbital angular momentum. *Sci. Bull.* **65**, 1334-1336 (2020).
19. Plick, W. N., & Mario K. Physical meaning of the radial index of Laguerre-Gauss beams. Phys. Rev. A **92**, 063841 (2015).
